# Medication Adherence Among Hypertensive Patients in Bangladesh: A Nationwide Analysis of Gender and Urban–Rural Disparities

**DOI:** 10.1155/ijhy/9886514

**Published:** 2026-05-30

**Authors:** Mahmila Sanjana Mim, Md. Muddasir Hossain Akib, Bikash Pal, Md. Lutfor Rahaman

**Affiliations:** ^1^ Department of Statistics, University of Dhaka, Dhaka, Bangladesh, du.ac.bd; ^2^ Southeast Business School, Southeast University, Dhaka, Bangladesh, seu.edu.bd

**Keywords:** Bangladesh, BDHS 2022, binary logistic regression, gender differences, hypertension, medication adherence, urban–rural disparities

## Abstract

Hypertension remains a major public health concern in Bangladesh, yet comprehensive nationwide studies examining prescription‐based medication adherence are limited. To address this gap and understand adherence patterns for effective hypertension management, this study utilized secondary data from the Bangladesh Demographic and Health Survey (BDHS) 2022. A total of 1597 hypertensive individuals aged 18 years or older (670 urban, 927 rural) were included after excluding cases with missing values. Medication adherence was defined by self‐reported use of prescribed antihypertensive drugs among those identified with hypertension (SBP ≥ 140 mmHg and/or DBP ≥ 90 mmHg or currently taking medication). Univariate, bivariate, and multilevel binary logistic regression analyses were conducted to identify key factors influencing adherence. Findings revealed that 83.2% of participants were adherent to medication. Adherence varied significantly by gender, wealth status, and diabetes comorbidity. In urban males, those from middle and rich households were 90.4% and 86.8% less likely to be adherent than poor counterparts, while nondiabetic men were over four times more likely to be adherent (AOR: 4.459, 95% CI = (1.371, 13.224), *p* value = 0.013). Among rural females, higher wealth status increased adherence (AOR: 1.998, 95% CI = (1.221, 3.268), *p* value = 0.006), but those with primary or secondary education showed lower odds of adherence. Urban females from rich households had significantly higher odds of adherence (AOR: 4.759, 95% CI = (1.838, 12.335), *p* value = 0.002). These findings highlight the substantial gender and urban–rural disparities in hypertension medication adherence in Bangladesh. Targeted policies, such as educational outreach for rural women, expanded diabetes screening in urban men, and subsidized medication access in rural areas, are urgently needed to improve hypertension management.

## 1. Introduction

Excessive blood pressure, often known as hypertension, is a widespread and serious health condition characterized by consistently elevated pressure in the blood vessels [1]. The leading risk factor for cardiovascular disease (CVD) is hypertension, which is responsible for 50% of coronary heart disease cases and nearly two‐thirds of the global burden of cerebrovascular disease [2, 3]. This “silent killer” is also linked to several other conditions, including stroke, kidney failure, disability, and early mortality [4–6]. Over 1.3 billion people worldwide suffer from hypertension, which causes more than 7 million fatalities yearly, according to the World Health Organization (WHO) [7]. In recent years, the situation has become increasingly alarming. Nearly half of adults with hypertension are unaware of their condition, and less than half receive proper diagnosis and treatment. A heavy economic toll is evident through substantial medical costs and diminished productivity levels [6]. Furthermore, hypertension intersects with several Sustainable Development Goals (SDGs), especially SDG 3 (good health and well‐being). Tackling hypertension is vital for reducing noncommunicable diseases (NCDs) and improving mental health. It also connects with SDG 2 (zero hunger) through the importance of a balanced diet, SDG 1 (no poverty) and SDG 10 (reduced inequalities) due to its higher prevalence in low‐ and middle‐income populations, and SDG 4 (quality education) by emphasizing the importance of health literacy [8, 9].

Despite being a worldwide issue, the prevalence of hypertension has significantly increased, particularly in low‐ and middle‐income countries (LMICs) [10]. According to the WHO, the number of people with hypertension has nearly doubled since 1990, reaching 1.28 billion adults aged 30–79 years worldwide in 2019 [11]. A large portion of this rise has occurred in LMICs, where two‐thirds reside [7]. Aging and population growth are the primary causes of this rise [12]. Worrying projections suggest a 30% surge in global hypertension prevalence by 2025, with LMICs expected to shoulder the majority of this increase [13]. By 2040, the incidence of hypertension is predicted to increase significantly worldwide, with low‐income countries estimated to have the highest frequency [14].

Medication adherence is the extent to which patients follow their prescribed medication regimens to ensure that they take the correct dose at the right time and for the recommended duration [15]. Maintaining high medication adherence is essential for effectively managing chronic conditions. For individuals with hypertension, adhering to their medication regimen is important for managing blood pressure and reducing the risk of complications like heart attacks, strokes, and kidney failure [16]. Patients who take their medications as directed have a higher chance of reaching and maintaining their desired blood pressure levels, which improves their health [17]. Unfortunately, numerous patients face difficulties maintaining adherence due to different factors [15, 18–22]. Besides, urban–rural disparities exist in hypertension management. Rural regions often struggle with limited access to healthcare facilities, lower health literacy, and socioeconomic challenges, resulting in poorer medication adherence [23]. Moreover, women generally exhibit lower medication adherence rates compared to men [24]. This disparity can be attributed to different factors, such as health literacy, socioeconomic status, and cultural norms [24, 25].

In LMICs, only approximately 42% of adults with hypertension receive a diagnosis and treatment, and a mere 21% manage to keep their condition under control [7]. Major risk factors that are responsible for the high prevalence of LMICs encompass unhealthy diets characterized by high salt intake and low consumption of fruits and vegetables, along with physical inactivity, tobacco and alcohol use, and obesity [26]. Environmental factors such as air pollution also play a significant role [26]. LMICs also grapple with challenges like insufficient healthcare resources, a low ratio of physicians to patients, and restricted access to essential medications [26]. Moreover, in LMICs, while hypertension is more prevalent in urban areas compared to rural areas, the rate of increase in hypertension prevalence is higher in rural areas [27]. The prevalence rates in high‐income countries (HICs) are generally more consistent, owing to the robust healthcare infrastructure [6]. Awareness, diagnosis, and treatment rates are notably higher in HICs than in LMICs [7]. Nevertheless, even in HICs, fewer than 50% of adults with hypertension manage to keep their condition under control [28]. The rising prevalence underscores significant disparities between HICs and LMICs [29]. For instance, in South Asia, the rate of hypertension is escalating swiftly, with populations in LMICs like Bangladesh being especially at risk [30–32].

Previously, hypertension was relatively uncommon in Bangladesh, as prevalence rates remained low and public health efforts primarily targeted communicable diseases [33]. However, the present situation reflects a significant epidemiological shift toward NCDs, as hypertension emerges as a major health concern [33–37]. Studies indicate that the prevalence of uncontrolled hypertension in Bangladesh varies between 25% and 50% [35, 38, 39]. In Bangladesh, prevalent risk factors for hypertension include excess body weight, sedentary habits, poor diet, high salt consumption, diabetes, use of tobacco and alcohol, air pollution, and lack of physical activity [37, 40, 41]. Enhancing medication adherence is vital for achieving better blood pressure control and alleviating the burden of hypertension‐related complications [42]. Despite the growing evidence on the significance of medication adherence, follow‐up history, and the status of multimorbidity in the comprehensive management of hypertension, Bangladesh has yet to address or incorporate these aspects into its national guidelines for hypertension management [43].

Numerous studies in Bangladesh have scrutinized the prevalence, risk factors, and awareness of hypertension, which have predominantly focused on hospital‐based settings or specific population groups [37, 44–46]. Notably, Khanam et al. (2014) conducted a rigorous population‐based study in rural Bangladesh using BDHS‐associated data, offering important insights into treatment adherence and its socioeconomic and provider‐related determinants. While their work laid a strong foundation, it did not comprehensively examine prescription‐based adherence across both urban and rural contexts or explore gender disparities at the national level [46].

To address these gaps, the present study draws on the most recent Bangladesh Demographic and Health Survey (BDHS) data to conduct a nationwide analysis of antihypertensive medication adherence. It specifically focuses on prescription‐based adherence and investigates gender and urban–rural disparities that remain underexplored in existing literature. By doing so, the study aims to inform more inclusive and evidence‐based strategies for improving hypertension management and patient outcomes in Bangladesh.

## 2. Methods

### 2.1. Study Design and Data Source

**FIGURE 1:**
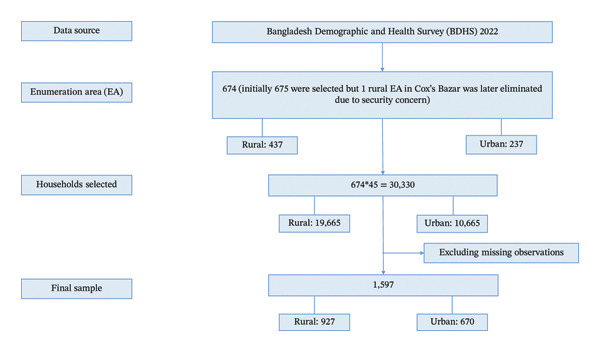
Flowchart of the sample selection process.

In this study, secondary data extracted from the BDHS 2022 were utilized. The survey was conducted under the authority of the National Institute of Population Research and Training (NIPORT), Ministry of Health and Family Welfare (MoHFW), Medical Education and Family Welfare Division. The BDHS 2022 sample followed a two‐stage stratified selection process. First, 675 enumeration areas (EAs) were selected based on their size, with independent selections within each stratum. Among them, 438 EAs were selected from rural regions and 237 from urban regions of Bangladesh. In the second stage, 45 households per cluster were systematically chosen from the new household listings. The survey targeted all ever‐married women aged 15–49 who were either usual residents or had stayed the night before the survey in the selected households. Based on this design, a total of 30,330 residential households were selected, with 19,665 from rural areas and 10,665 from urban areas. Biomarker data, such as blood pressure, fasting plasma blood glucose, and height‐weight measurements, were available for all women and males aged 18 and older in one‐fourth of the randomly chosen households. For measuring blood pressure and blood glucose, 6853 men and 8156 women at least 18 years old were eligible. 95% of women and 91% of men eligible for the study had their blood pressure measured, while 92% of women and 89% of men had their blood glucose measured [47]. After excluding missing values and considering only the hypertensive patients, our final dataset included 1597 observations (927 in rural and 670 in urban areas). Figure 1 presents the process used for sample selection.

### 2.2. Outcome Variable

The outcome variable considered in this study is the medication adherence status of hypertensive patients. Individuals were identified to have hypertension if their systolic blood pressure (SBP) was ≥ 140 mmHg and/or their diastolic blood pressure (DBP) was ≥ 90 mmHg or if they were taking antihypertensive medication during the survey [7, 47, 48]. All hypertensive study participants were asked if they had been prescribed medication to control hypertension. If they responded “yes”, a follow‐up question inquired whether they were taking the prescribed antihypertensive or blood‐pressure‐lowering medication. If they responded “yes” again, they were considered adherent. Otherwise, they were classified as nonadherent.

### 2.3. Covariates

All the explanatory variables considered in this study are categorical, having at least two categories (independent groups). The following covariates have been considered in this study: age (18–29, 30–39, 40–49, 50–59, 60–69, and 70+), sex (male and female), place of residence (urban and rural), division (Barisal, Chittagong, Dhaka, Khulna, Mymensingh, Rajshahi, Rangpur, and Sylhet) [49], current marital status (currently married and not currently married), education level (no education, primary, secondary, and higher), wealth index (poor, middle, and rich), body mass index (underweight, normal, overweight, and obese), and presence of diabetes (yes and no). The BDHS wealth index is an asset‐based measure of household socioeconomic status and is not based on household income [47]. Households were classified into five quintiles (poorest, poorer, middle, richer, and richest), which were regrouped into three categories in this study: poor (poorest and poorer), middle, and rich (richer and richest) [50–53]. Body mass index (BMI) is classified as underweight if it is less than 18.5, normal if it is between 18.5 and 24.9, overweight if it is between 25 and 29.9, and obese if it is 30 or higher [54, 55]. The BDHS 2022 survey assessed diabetes prevalence among individuals aged 18 and above by measuring fasting plasma glucose levels. Respondents were classified as diabetic if their blood glucose was 7.0 mmol/L or higher or if they were taking prescribed diabetes medication [47, 56].

### 2.4. Statistical Analyses

The statistical analyses were conducted using SPSS version 20 and R programming version 4.1.3. The analytical approach began with univariate analysis, which provided a comprehensive overview of individual variable distributions to allow for an initial assessment of their characteristics and potential data trends. This was followed by bivariate analysis to explore the relationships between selected independent variables and the outcome variable, medication adherence status of hypertensive patients. Specifically, the chi‐square test was employed to assess associations between categorical variables and adherence status, identifying statistically significant relationships. This nonparametric test is widely used in public health research to determine whether observed differences in proportions are statistically significant [57, 58]. To further assess the determinants of adherence, binary logistic regression was applied, offering a predictive model that estimates the likelihood of adherence based on multiple predictor variables [59]. Separate logistic regression models were fitted for each subgroup (urban and rural populations, stratified by sex), resulting in a total of six models. Model fit was assessed using the Nagelkerke pseudo *R* ^2^, an appropriate measure for logistic regression. Variables found to be statistically significant in the bivariate analysis were included in the respective multivariable models. Nonsignificant variables were excluded to obtain parsimonious models. Since the outcome variable was binary, classifying patients as either adherent or nonadherent, binary logistic regression method was well‐suited for analyzing the influence of independent variables on adherence patterns. The regression analysis also accounted for sample weighting, stratification, and clustering, ensuring that the results were representative of the population and accounted for complex survey design effects [59, 60]. Multicollinearity among the independent variables was assessed using the variance inflation factor (VIF), a widely used diagnostic measure in regression analysis [61]. All VIF values were below the commonly accepted threshold of 10, indicating no evidence of problematic multicollinearity. Tolerance, defined as the reciprocal of VIF, was not reported separately as it provides equivalent information [62]. Odds ratios were calculated to quantify the strength and direction of associations, enabling a deeper understanding of how demographic, socioeconomic, and health‐related factors influence medication adherence.

## 3. Results

### 3.1. Univariate Analysis

Table 1 displays the frequency, valid percentage, and cumulative percentage of each study variable. It reveals that 83.2% of the respondents adhered to medication, while 16.8% did not. The sample was composed of 32.7% males and 67.3% females. Regarding residence, 42.0% lived in urban areas, and 58.0% resided in rural areas. The highest proportion of respondents hailed from Rajshahi (14.8%), and the lowest from Mymensingh (9.1%). Participants aged 50–59 years formed the majority (25.9%), with those aged 18–29 years being the smallest group (4.0%). Education levels showed 35.0% with no education, 24.6% with primary education, 26.5% with secondary education, and 13.8% with higher education. Wealth distribution indicated that 57.7% were categorized as rich, 17.0% as middle‐class, and 25.3% as poor. Marital status data revealed that more than three‐fourth of the respondents were currently married, and 23.8% were not. In terms of health, 31.1% had diabetes, while 68.9% did not. BMI categories included 8.0% underweight, 30.4% normal weight, 27.7% overweight, and 35.6% obese.

**TABLE 1:** Univariate analysis of dependent and independent variables.

Variable	Frequency	Valid percent (%)	Cumulative percent (%)
*Medical adherence*			
Yes	1329	83.2	83.2
No	268	16.8	100.0

*Sex*			
Male	523	32.7	32.7
Female	1074	67.3	100.0

*Place of residence*			
Urban	670	42.0	42.0
Rural	927	58.0	100.0

*Division*			
Sylhet	219	13.7	13.7
Chattogram	227	14.2	27.9
Dhaka	192	12.0	39.9
Khulna	230	14.4	54.4
Mymensingh	145	9.1	63.4
Rajshahi	236	14.8	78.2
Rangpur	189	11.8	90.0
Barishal	159	10.0	100.0

*Age group*			
18–29	64	4.0	4.0
30–39	199	12.5	16.5
40–49	337	21.1	37.6
50–59	414	25.9	63.5
60–69	363	22.7	86.2
70+	220	13.8	100.0

*Education level*			
No education	559	35.0	35.0
Primary	393	24.6	59.6
Secondary	424	26.5	86.1
Higher	221	13.8	100.0

*Wealth index*			
Poor	404	25.3	25.3
Middle	271	17.0	42.3
Rich	922	57.7	100.0

*Marital status*			
Currently married	1217	23.8	23.8
Not currently married	380	76.2	100.0

*Diabetes*			
Yes	497	31.1	31.1
No	1100	68.9	100.0

*Body mass index*			
Underweight	127	8.0	8.0
Normal weight	709	44.0	52.0
Overweight	570	35.7	87.7
Obese	191	12.0	100.0

### 3.2. Bivariate Analysis

Table 2 presents medication adherence patterns among hypertensive males, showing 11.2% in urban Sylhet and 14.5% in rural Sylhet. Education level significantly influenced rural males, with adherence rates of 18.9% among those with higher education, compared to 28.2% for secondary, 23.3% for primary, and 29.5% for those with no education. Diabetes status also revealed differences, with diabetic males adhering at 40.8% in urban areas and 24.7% in rural areas. *p* values confirmed significant associations between division, education and diabetes status with adherence among males.

**TABLE 2:** Bivariate frequency distribution of male’s medication adherence status among the different categories of selected covariates.

Covariates	Male							
	Urban				Rural			
	Adherent	Nonadherent	COR (95% CI)	*p* value	Adherent	Nonadherent	COR (95% CI)	*p* value
*Division*				*0.632*				***0.012***
Dhaka (Ref.)	23 (11.2%)	3 (7.9%)	1.000 —		33 (14.5%)	5 (9.6%)	1.000 —	
Chattogram	24 (11.7%)	5 (13.2%)	1.149 (0.409,3.226)		35 (15.4%)	2 (3.8%)	0.219 (0.051, 0.943)	
Sylhet	36 17.5%)	8 (21.1%)	0.682 (0.194, 2.395)		24 (10.6%)	5 (9.6%)	0.625 (0.232, 1.688)	
Khulna	41 (19.9%)	5 (13.2%)	0.610 (0.224, 1.659)		39 (17.2%)	9 (17.3%)	1.009 (0.455, 2.239)	
Mymensingh	15 (7.3%)	1 (2.6%)	0.344 (0.044, 2.686)		18 (7.9%)	2 (3.8%)	0.464 (0.104, 2.067)	
Rajshahi	27 (13.1%)	8 (21.1%)	1.768 (0.734, 4.256)		26 (11.5%)	12 (23.1%)	2.319 (1.081, 4.977)	
Rangpur	26 (12.6%)	6 (15.8%)	1.298 (0.495, 3.404)		29 (12.8%)	11 (21.2%)	1.832 (0.847, 3.961)	
Barishal	14 (6.8%)	2 (5.3%)	0.762 (0.166, 3.497)		23 (10.1%)	6 (11.5%)	1.157 (0.446, 3.003)	

*Age group*				*0.831*				*0.770*
18–29 (Ref.)	7 (3.4%)	4 (10.5%)	1.000 —		3 (1.3%)	4 (7.7%)	1.000 —	
30–39	20 (9.7%)	4 (10.5%)	1.094 (0.352, 3.401)		21 (9.3%)	4 (7.7%)	0.817 (0.268, 2.491)	
40–49	43 (20.9%)	11 (28.9%)	1.544 (0.710, 3.360)		28 (12.3%)	9 (17.3%)	1.488 (0.655, 3.378)	
50–59	62 (30.1%)	9 (23.7%)	0.721 (0.322, 1.612)		58 (25.6%)	12 (23.1%)	0.874 (0.429, 1.779)	
60–69	47 (22.8%)	5 (13.2%)	0.513 (0.189, 1.387)		67 (29.5%)	12 (23.1%)	0.716 (0.354, 1.450)	
70+	27 (13.1%)	5 (13.2%)	1.004 (0.361, 2.797)		50 (22.0%)	11 (21.2%)	0.950 (0.455, 1.982)	

*Education level*				*0.566*				***0.004***
No education (Ref.)	28 (13.6%)	5 (13.2%)	1.000 —		67 (29.5%)	24 (46.2%)	1.000 —	
Primary	30 (14.6%)	7 (18.4%)	1.325 (0.535, 3.281)		53 (23.3%)	10 (19.2%)	0.782 (0.367, 1.663)	
Secondary	67 (32.5%)	14 (36.8%)	1.210 (0.589, 2.488)		64 (28.2%)	15 (28.8%)	1.033 (0.530, 2.010)	
Higher	81 (39.3%)	12 (31.6%)	0.712 (0.340, 1.491)		43 (18.9%)	3 (5.8%)	0.262 (0.078, 0.880)	

*Wealth index*				*0.653*				*0.082*
Poor (Ref.)	15 (7.3%)	1 (2.6%)	1.000 —		72 (31.7%)	22 (42.3%)	1.000 —	
Middle	17 (8.3%)	5 (13.2%)	1.684 (0.582, 4.879)		47 (20.7%)	12 (23.1%)	1.149 (0.559, 2.362)	
Rich	174 (84.5%)	32 (84.2%)	0.981 (0.379, 2.536)		108 (47.6%)	18 (34.6%)	0.583 (0.311, 1.093)	

*Marital status*				*0.718*				*0.051*
Not currently married (Ref.)	193 (93.7%)	35 (92.1%)	1.000 —		214 (94.3%)	45 (86.5%)	1.000 —	
Currently married	13 (6.3%)	3 (7.9%)	0.786 (0.213, 2.901)		13 (5.7%)	7 (13.5%)	0.391 (0.148, 1.034)	

*Diabetes*				***0.009***				***0.003***
Yes (Ref.)	84 (40.8%)	7 (18.4%)	1.000 —		56 (24.7%)	3 (5.8%)	1.000 —	
No	122 (59.2%)	31 (81.6%)	0.328 (0.138, 0.780)		171 (75.3%)	49 (94.2%)	0.187 (0.056, 0.623)	

*Body mass index*				*0.604*				*0.078*
Underweight (Ref.)	11 (5.3%)	0 (0.0%)	1.000 —		22 (9.7%)	8 (15.4%)	1.000 —	
Normal weight	93 (45.1%)	16 (42.1%)	0.884 (0.439, 1.780)		136 (59.9%)	28 (53.8%)	0.781 (0.426, 1.432)	
Overweight	82 (39.8%)	20 (52.6%)	1.680 (0.838, 3.367)		60 (26.4%)	16 (30.8%)	1.237 (0.640, 2.390)	
Obese	20 (9.7%)	2 (5.3%)	0.517 (0.116, 2.308)		9 (4.0%)	0 (0.0%)	— —	

*Note:* The bold values represent statistically significant *p* values, indicating that the associations observed are unlikely to be due to chance.

Table 3 highlights female adherence, with 13.3% in urban Sylhet and 16.5% in rural Sylhet, again showing higher rural compliance. Unlike males, education did not significantly affect adherence among females. Wealth disparities were evident, with adherence in the rich group at 81.0% in urban areas compared to 43.8% in rural areas. Diabetes status also showed meaningful differences, with adherence rates of 41.0% in urban areas and 28.2% in rural areas. *p* values demonstrated significant associations between wealth and adherence for females, while diabetes showed notable but not statistically significant effects.

**TABLE 3:** Bivariate frequency distribution of female’s medication adherence status among the different categories of selected covariates.

Covariates	Female							
	Urban				Rural			
	Adherent	Nonadherent	COR (95% CI)	*p* value	Adherent	Nonadherent	COR (95% CI)	*p* value
*Division*				*0.977*				***0.015***
Dhaka (Ref.)	49 (13.3%)	7 (12.1%)	1.000 —		87 (16.5%)	12 (10.0%)	1.000 —	
Chattogram	65 (17.7%)	13 (22.4%)	1.347 (0.687, 2.639)		74 (14.0%)	9 (7.5%)	0.497 (0.242, 1.024)	
Sylhet	52 (14.1%)	5 (8.6%)	0.894 (0.384, 2.081)		50 (9.5%)	12 (10.0%)	0.563 (0.297, 1.067)	
Khulna	45 (12.2%)	6 (10.3%)	0.828 (0.336, 2.039)		75 (14.2%)	10 (8.3%)	0.549 (0.275, 1.097)	
Mymensingh	29 (7.9%)	8 (13.8%)	1.870 (0.810, 4.320)		49 (9.3%)	23 (19.3%)	2.318 (1.349, 3.983)	
Rajshahi	58 (15.8%)	7 (12.1%)	0.734 (0.317, 1.696)		72 (13.6%)	26 (21.7%)	1.752 (1.062, 2.889)	
Rangpur	36 (9.8%)	8 (13.8%)	1.476 (0.649, 3.356)		57 (10.8%)	16 (13.3%)	1.271 (0.702, 2.302)	
Barishal	34 (9.2%)	4 (6.9%)	0.728 (0.248, 2.132)		64 (12.1%)	12 (10.0%)	0.806 (0.420, 1.545)	

*Age group*				*0.754*				*0.318*
18–29 (Ref.)	16 (4.3%)	4 (6.9%)	1.000 —		21 (4.0%)	5 (4.2%)	1.000 —	
30–39	55 (14.9%)	13 (22.4%)	1.644 (0.833, 3.247)		59 (11.2%)	23 (19.2%)	1.885 (1.110, 3.199)	
40–49	96 (26.1%)	17 (29.3%)	1.175 (0.637, 2.165)		106 (20.1%)	27 (22.5%)	1.156 (0.716, 1.865)	
50–59	94 (25.5%)	7 (12.1%)	0.400 (0.176, 0.912)		132 (25.0%)	40 (33.3%)	1.500 (0.978, 2.300)	
60–69	69 (18.8%)	12 (20.7%)	1.130 (0.569, 2.247)		136 (25.8%)	15 (12.5%)	0.412 (0.232, 0.732)	
70+	38 (10.3%)	5 (8.6%)	0.819 (0.309, 2.175)		74 (14.0%)	10 (8.3%)	0.558 (0.279, 1.115)	

*Education level*				*0.231*				*0.151*
No education (Ref.)	108 (29.3%)	23 (39.7%)	1.000 —		257 (48.7%)	47 (39.2%)	1.000 —	
Primary	92 (25.0%)	11 (19.0%)	0.702 (0.350, 1.411)		145 (27.5%)	45 (37.5%)	1.585 (1.045, 2.403)	
Secondary	115 (31.2%)	16 (27.6%)	0.838 (0.452, 1.553)		106 (20.1%)	27 (22.5%)	1.156 (0.716, 1.865)	
Higher	53 (14.4%)	8 (13.8%)	0.951 (0.427, 2.119)		20 (3.8%)	1 (0.8%)	0.213 (0.028, 1.606)	

*Wealth index*				***≤ 0.001***				***≤ 0.001***
Poor (Ref.)	27 (7.3%)	11 (19.0%)	1.000 —		195 (36.9%)	61 (50.8%)	1.000 —	
Middle	43 (11.7%)	17 (29.3%)	3.134 (1.638, 5.995)		102 (19.3%)	28 (23.3%)	1.271 (0.790, 2.044)	
Rich	298 (81.0%)	30 (51.7%)	0.252 (0.141, 0.448)		231 (43.8%)	31 (25.8%)	0.448 (0.287, 0.698)	

*Marital status*				*0.860*				*0.054*
Not currently married (Ref.)	258 (70.1%)	40 (69.0%)	1.000 —		343 (65.0%)	89 (74.2%)	1.000 —	
Currently married	110 (29.9%)	18 (31.0%)	0.947 (0.520,1.725)		185 (35.0%)	31 (25.8%)	1.548 (0.991,2.419)	

*Diabetes*				*0.487*				*0.145*
Yes (Ref.)	151 (41.0%)	21 (36.2%)	1.000 —		149 (28.2%)	26 (21.7%)	1.000 —	
No	217 (59.0%)	37 (63.8%)	0.816 (0.459, 1.449)		379 (71.8%)	94 (78.3%)	0.704 (0.438, 1.130)	

*Body mass index*				*0.104*				*0.755*
Underweight (Ref.)	14 (3.8%)	3 (5.2%)	1.000 —		56 (10.6%)	13 (10.8%)	1.000 —	
Normal weight	107 (29.1%)	22 (37.9%)	1.491 (0.838, 2.652)		250 (47.3%)	57 (47.5%)	1.006 (0.676, 1.496)	
Overweight	159 (43.2%)	27 (46.6%)	1.145 (0.657, 1.996)		167 (31.6%)	39 (32.5%)	1.041 (0.681, 1.590)	
Obese	88 (23.9%)	6 (10.3%)	0.367 (0.153, 0.884)		55 (10.4%)	11 (9.2%)	0.868 (0.440, 1.713)	

*Note:* The bold values represent statistically significant *p* values, indicating that the associations observed are unlikely to be due to chance.

Table 4 shows combined adherence in Sylhet was 12.5% in urban areas and 15.9% in rural areas, reflecting a consistent rural advantage across genders. Age group did not significantly affect adherence, while education level showed limited influence overall. Wealth disparities were substantial, with adherence rates of 82.2% in urban areas and 44.9% in rural areas. Diabetes status also played a critical role, with overall adherence among diabetic patients at 40.9% in urban areas and 27.2% in rural areas. *p* values confirmed significant associations between both wealth and diabetes status with overall adherence, underscoring the importance of socioeconomic and health‐related factors in shaping medication compliance.

**TABLE 4:** Bivariate frequency distribution of overall medication adherence status among the different categories of selected covariates.

Covariates	Overall (combined male and female)							
	Urban				Rural			
	Adherent	Nonadherent	COR (95% CI)	*p* value	Adherent	Nonadherent	COR (95% CI)	*p* value
*Division*				*0.744*				***≤ 0.001***
Dhaka (Ref.)	88 (15.3%)	13 (13.5%)	1.000 —		120 (15.9%)	17 (9.9%)	1.000 —	
Chattogram	89 (15.5%)	18 (18.8%)	1.258 (0.718, 2.202)		109 (14.4%)	11 (6.4%)	0.405 (0.213, 0.771)	
Sylhet	72 (12.5%)	10 (10.4%)	0.811 (0.403, 1.632)		74 (9.8%)	17 (9.9%)	0.580 (0.339, 0.993)	
Khulna	86 (15.0%)	11 (11.5%)	0.734 (0.376, 1.433)		114 (15.1%)	19 (11.0%)	0.698 (0.417, 1.171)	
Mymensingh	44 (7.7%)	9 (9.4%)	1.246 (0.587, 2.643)		67 (8.9%)	25 (14.5%)	1.746 (1.067, 2.858)	
Rajshahi	85 (14.8%)	15 (15.6%)	1.065 (0.586, 1.936)		98 (13.0%)	38 (22.1%)	1.901 (1.252, 2.888)	
Rangpur	62 (10.8%)	14 (14.6%)	1.410 (0.755, 2.634)		86 (11.4%)	27 (15.7%)	1.449 (0.907, 2.313)	
Barishal	48 (8.4%)	6 (6.2%)	0.731 (0.304, 1.757)		87 (11.5%)	18 (10.5%)	0.897 (0.525, 1.535)	

*Age group*				*0.722*				*0.526*
18–29 (Ref.)	23 (4.0%)	8 (8.3%)	1.000 —		24 (3.2%)	9 (5.2%)	1.000 —	
30–39	75 (13.1%)	17 (17.7%)	1.432 (0.804, 2.551)		80 (10.6%)	27 (15.7%)	1.571 (0.980, 2.518)	
40–49	139 (24.2%)	28 (29.2%)	1.289 (0.798, 2.082)		134 (17.7%)	36 (20.9%)	1.227 (0.812, 1.852)	
50–59	156 (27.2%)	16 (16.7%)	0.536 (0.304, 0.945)		190 (25.2%)	52 (30.2%)	1.289 (0.895, 1.855)	
60–69	116 (20.2%)	17 (17.7%)	0.850 (0.484, 1.491)		203 (26.9%)	27 (15.7%)	0.506 (0.326, 0.787)	
70+	65 (11.3%)	10 (10.4%)	0.911 (0.450, 1.841)		124 (16.4%)	21 (12.2%)	0.708 (0.431, 1.161)	

*Education level*				*0.281*				*0.580*
No education (Ref.)	136 (23.7%)	28 (29.2%)	1.000 —		324 (42.9%)	71 (41.3%)	1.000 —	
Primary	122 (21.3%)	18 (18.8%)	0.855 (0.493, 1.482)		198 (26.2%)	55 (32.0%)	1.322 (0.923, 1.894)	
Secondary	182 (31.7%)	30 (31.2%)	0.979 (0.614, 1.560)		170 (22.5%)	42 (24.4%)	1.112 (0.755, 1.638)	
Higher	134 (23.3%)	20 (20.8%)	0.864 (0.509, 1.467)		63 (8.3%)	4 (2.3%)	0.262 (0.094, 0.729)	

*Wealth index*				***≤ 0.001***				***≤ 0.001***
Poor (Ref.)	42 (7.3%)	12 (12.5%)	1.000 —		267 (35.4%)	83 (48.3%)	1.000 —	
Middle	60 (10.5%)	22 (22.9%)	2.547 (1.475, 4.396)		149 (19.7%)	40 (23.3%)	1.232 (0.829, 1.832)	
Rich	472 (82.2%)	62 (64.6%)	0.394 (0.246, 0.630)		339 (44.9%)	49 (28.5%)	0.489 (0.341, 0.701)	

*Marital status*				*0.922*				*0.262*
Not currently married (Ref.)	123 (21.4%)	21 (21.9%)	1.000 —		557 (73.8%)	134 (77.9%)	1.000 —	
Currently married	451 (78.6%)	75 (78.1%)	0.974 (0.577, 1.644)		38 (22.1%)	198 (26.2%)	1.254 (0.844, 1.861)	

*Diabetes*				***0.029***				***0.005***
Yes (Ref.)	235 (40.9%)	28 (29.2%)	1.000 —		205 (27.2%)	29 (16.9%)	1.000 —	
No	339 (59.1%)	68 (70.8%)	0.594 (0.371, 0.951)		550 (72.8%)	143 (83.1%)	0.544 (0.354, 0.837)	

*Body mass index*				*0.093*				*0.245*
Underweight (Ref.)	25 (4.4%)	3 (3.1%)	1.000 —		78 (10.3%)	21 (12.2%)	1.000 —	
Normal weight	200 (34.8%)	38 (39.6%)	1.225 (0.786, 1.909)		386 (51.1%)	85 (49.4%)	0.934 (0.671, 1.301)	
Overweight	241 (42.0%)	47 (49.0%)	1.325 (0.859, 2.044)		227 (30.1%)	55 (32.0%)	1.093 (0.766, 1.561)	
Obese	108 (18.8%)	8 (8.3%)	0.392 (0.185, 0.833)		64 (8.5%)	11 (6.4%)	0.738 (0.380, 1.431)	

*Note:* The bold values represent statistically significant *p* values, indicating that the associations observed are unlikely to be due to chance.

**FIGURE 2:**
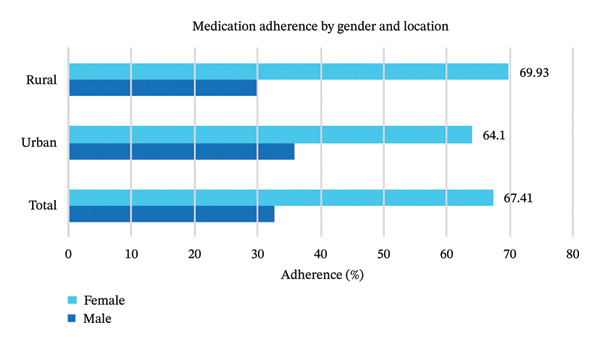
Patterns in medication adherence among hypertensive patients in Bangladesh, stratified by gender and location.

Figure 2 presents distinct patterns in medication adherence among hypertensive patients in Bangladesh, based on gender and place of residence. Females exhibit markedly higher adherence overall (67.41%) compared to males (32.59%), a pattern consistent across both urban and rural contexts. In urban areas, 64.1% of females follow their medication regimen, while the rate among males is 35.9%. The gap becomes more pronounced in rural settings, where 69.93% of females adhere to treatment compared to just 30.07% of males. These results highlight a pronounced gender disparity in adherence, with rural females exhibiting the highest adherence rates and rural males the lowest.

Prior to bivariate and multivariable analyses, multicollinearity among the independent variables was assessed using the VIF. The VIF values ranged from 1.006 to 1.163, indicating no evidence of problematic multicollinearity.

### 3.3. Binary Logistic Regression Analysis

The results presented in Table 5 indicate significant discrepancies in medication adherence status of hypertensive patients based on some selected variables. In urban males, those with a middle wealth index were 90.4% less likely, and those with a rich wealth index were 86.8% less likely to adhere to medication compared to those with a poor wealth index, while individuals without diabetes were 4.459 times likely to adhere. Among urban females, those with primary education were 59.2% less likely, and those with secondary education were 34.8% less likely to adhere compared to those with no education, whereas those with a rich wealth index were 99.8% more likely to adhere. This highlights a male–female discrepancy, where education played a more significant protective role in medication adherence for females, while wealth status and diabetes condition had a stronger influence in males. For the overall urban population, individuals without diabetes were 2.002 times likely to adhere to medication.

**TABLE 5:** Binary logistic regression estimates of the selected covariates.

Covariates	Male						Female						Overall (combined male and female)					
	Urban			Rural			Urban			Rural			Urban			Rural		
	β	*p* value	AOR (95% CI)	β	*p* value	AOR (95% CI)	β	*p* value	AOR (95% CI)	β	*p* value	AOR (95% CI)	β	*p* value	AOR (95% CI)	β	*p* value	AOR (95% CI)
Intercept	2.564	**0.021**	12.988 (1.502, 112.345)	0.784	0.229	2.190 (0.610, 7.867)	0.918	0.113	2.504 (0.806, 7.779)	1.471	**≤ 0.001**	4.354 (1.969, 9.630)	1.025	**0.033**	2.787 (1.093, 7.110)	1.243	**≤ 0.001**	3.466 (1.854, 6.484)

*Division*																		
Dhaka (Ref.)	—	—	—	—	—	—	—	—	—	—	—	—	—	—	—	—	—	—
Chattogram	0.526	0.451	1.692 (0.431, 6.647)	1.273	0.150	3.572 (0.632, 20.214)	−0.166	0.779	0.847 (0.266, 2.699)	0.657	0.198	1.929 (0.709, 5.247)	0.069	0.868	1.071 (0.474, 2.421)	−0.454	0.070	0.635 (0.389, 1.037)
Sylhet	1.292	0.086	3.640 (0.837, 15.833)	0.460	0.535	1.584 (0.370, 6.785)	0.272	0.692	1.313 (0.341, 5.058)	0.555	0.214	1.742 (0.726, 4.184)	0.625	0.227	1.868 (0.679, 5.146)	0.539	0.139	1.714 (0.839, 3.499)
Khulna	0.498	0.455	1.645 (0.446, 6.077)	−0.206	0.758	0.814 (0.219, 3.028)	−0.365	0.597	0.694 (0.179, 2.692)	0.647	0.180	1.910 (0.742, 4.918)	0.114	0.806	1.121 (0.453, 2.770)	0.355	0.356	1.426 (0.671, 3.032)
Mymensingh	1.194	0.256	3.300 (0.421, 25.865)	0.873	0.498	2.394 (0.191, 29.969)	−0.638	0.352	0.528 (0.138, 2.022)	−0.499	0.296	0.607 (0.238, 1.548)	−0.125	0.804	0.882 (0.328, 2.376)	−0.242	0.542	0.785 (0.360, 1.711)
Rajshahi	−0.854	0.204	0.426 (0.114, 1.586)	−0.748	0.262	0.473 (0.128, 1.751)	0.320	0.629	1.377 (0.375, 5.049)	−0.449	0.306	0.638 (0.270, 1.508)	−0.099	0.815	0.906 (0.396, 2.071)	−0.516	0.142	0.597 (0.300, 1.189)
Rangpur	0.047	0.943	1.048 (0.285, 3.852)	−0.639	0.359	0.528 (0.135, 2.072)	−0.166	0.788	0.847 (0.253, 2.835)	−0.026	0.956	0.974 (0.383, 2.475)	−0.008	0.987	0.992 (0.373, 2.637)	−0.215	0.567	0.807 (0.386, 1.686)
Barishal	0.456	0.739	1.578 (0.108, 23.099)	−0.049	0.947	0.952 (0.222, 4.077)	0.959	0.247	2.609 (0.516, 13.196)	0.501	0.293	1.650 (0.649, 4.200)	0.812	0.371	2.252 (0.38, 13.367)	0.324	0.412	1.383 (0.637, 3.003)

*Education level*																		
No education (Ref.)	—	—	—	—	—	—	—	—	—	—	—	—	—	—	—	—	—	—
Primary	−0.054	0.955	0.947 (0.147, 6.117)	0.761	0.110	2.140 (0.844, 5.422)	−0.290	0.575	0.748 (0.271, 2.064)	−0.897	**0.002**	0.408 (0.231, 0.720)	−0.248	0.641	0.780 (0.275, 2.215)	−0.454	0.070	0.635 (0.389, 1.037)
Secondary	0.448	0.534	1.565 (0.380, 6.447)	0.308	0.422	1.361 (0.640, 2.893)	−0.274	0.509	0.760 (0.337, 1.715)	−0.715	**0.021**	0.489 (0.267, 0.896)	−0.079	0.830	0.924 (0.450, 1.898)	−0.383	0.118	0.682 (0.423, 1.101)
Higher	0.874	0.274	2.396 (0.501, 11.483)	1.199	0.065	3.317 (0.933, 11.774)	−0.639	0.280	0.528 (0.166, 1.681)	1.167	0.263	3.212 (0.415, 24.838)	−0.136	0.750	0.873 (0.377, 2.021)	0.716	0.187	2.046 (0.706, 5.936)

*Wealth index*																		
Poor (Ref.)	—	—	—	—	—	—	—	—	—	—	—	—	—	—	—	—	—	—
Middle	−2.342	**0.049**	0.096 (0.010, 0.925)	0.413	0.397	1.511 (0.580, 3.936)	0.045	0.930	1.046 (0.385, 2.841)	0.028	0.921	1.028 (0.594, 1.778)	−0.367	0.421	0.693 (0.284, 1.693)	0.165	0.487	1.179 (0.740, 1.879)
Rich	−2.028	**0.044**	0.132 (0.018, 0.976)	0.240	0.552	1.271 (0.576, 2.803)	1.560	**0.002**	4.759 (1.838, 12.335)	0.692	**0.006**	1.998 (1.221, 3.268)	0.721	0.079	2.056 (0.921, 4.591)	0.519	**0.011**	1.680 (1.130, 2.500)

*Diabetes*																		
Yes (Ref.)	—	—	—	—	—	—	—	—	—	—	—	—	—	—	—	—	—	—
No	1.449	**0.013**	4.259 (1.371, 13.224)	1.215	0.071	3.370 (0.904, 12.549)	0.292	0.471	1.339 (0.605, 2.966)	0.275	0.303	1.317 (0.779, 2.226)	0.694	**0.039**	2.002 (1.040, 3.848)	0.490	**0.042**	1.632 (1.018, 2.615)

*Note:* Model Nagelkerke *R* ^2^ values: urban male = 0.175; rural male = 0.183; urban female = 0.120; rural female = 0.116; urban overall = 0.072; rural overall = 0.095. In Table 5, the bold values represent statistically significant *p* values, indicating that the associations observed are unlikely to be due to chance.

In rural areas, those with a rich wealth index were 68% more likely to adhere compared to those with a poor wealth index. Similarly, rural individuals without diabetes were 63.2% more likely to adhere to medication. Moreover, rural females having a rich wealth index were 4.759 times likely to adhere to medication.

The Nagelkerke *R* ^2^ values indicate that, while the models identified significant predictors of medication adherence, a considerable proportion of the variability remains unexplained, reflecting the multifactorial nature of adherence behavior.

## 4. Discussion

The study shows clear differences in medication adherence among hypertensive patients in Bangladesh based on gender and urban–rural location. The analysis indicates that wealth status, education level, and diabetes all play roles in how well patients follow their medication routines.

Among urban males, individuals in the middle and high wealth groups were less likely to take their medication regularly compared to those in lower wealth groups. This finding diverges from conventional literature, which often associates higher income with improved access and adherence. However, emerging studies suggest that in urban contexts, affluence may correlate with reduced reliance on routine treatment due to perceived self‐efficacy, fragmented care‐seeking behavior, or preference for alternative therapies. For example, Islam et al. [63] and Xu et al. [64] report that wealthier individuals may exhibit lower adherence due to overconfidence in self‐management and exposure to nonconventional health practices. While this contrasts with studies by Arbuckle et al. [65] and Macquart de Terline et al. [66], which link wealth to better adherence in resource‐constrained settings, the discrepancy may reflect behavioral nuances specific to urban Bangladesh. Nonetheless, we acknowledge that these interpretations are speculative and not directly supported by empirical data in this study. Future research incorporating behavioral and qualitative dimensions is needed to validate these hypotheses.

In addition, urban males without diabetes were more likely to adhere to their medication schedules, suggesting that managing multiple health conditions adds complexity and may reduce consistent medication use. This finding mirrors previous research indicating that individuals without comorbid conditions, such as diabetes, may exhibit better medication adherence due to lower treatment complexity and reduced pill burden. Studies by Foley et al. [67] and Aspen RxHealth [68] similarly report that multimorbidity complicates self‐management through fragmented care, polypharmacy, and increased healthcare demands—factors likely contributing to reduced adherence among individuals with multiple conditions.

Furthermore, among rural females, individuals with primary or secondary education exhibited lower levels of medication adherence compared to those without formal education, which challenges the commonly held belief that higher educational attainment uniformly promotes better health behaviors. This paradox echoes findings by Wilhelmsen et al., who noted that individuals with moderate education may be exposed to conflicting health information or hold belief systems that complicate adherence [69]. While higher education is generally associated with improved health literacy and medication practices, as shown in other studies [70, 71], the inconsistency observed here may reflect unique cultural and contextual dynamics in rural Bangladesh. For example, educated women may face competing health narratives, social expectations, or limited engagement with formal healthcare systems, which could undermine adherence. These findings underscore the need for qualitative research to explore how education intersects with gender roles, health beliefs, and rural social structures. Notably, rural females in the higher wealth category were significantly more likely to adhere to their medication regimen, suggesting that financial resources may mitigate some of the barriers linked to education—a pattern consistent with previous studies [72, 73].

In urban populations, wealth was the main factor influencing adherence among females. Women from wealthier urban households were significantly more likely to follow their medication routines, underscoring the importance of financial capacity in shaping adherence behavior. This pattern reflects the challenges faced in urban areas, where economic disparities continue to determine access to consistent healthcare and medication. The finding supports previous studies that demonstrate the role of financial resources in sustaining medication adherence in resource‐constrained settings [46, 74].

In rural populations overall, both wealth and diabetes status significantly influenced adherence. The presence of diabetes was associated with reduced adherence, underscoring the difficulties of managing multiple health conditions when resources are scarce. This dual effect aligns with previous research on hypertensive patients in rural Bangladesh. For instance, Khanam et al. reported that coexisting conditions adversely affect medication adherence in these settings [46]. Similarly, Mamaghani et al. observed that in deprived rural areas, multiple comorbidities further complicate treatment adherence [75]. In the overall urban population, diabetes status was the main factor influencing adherence. Individuals without diabetes were significantly more likely to adhere, underscoring the burden of comorbid conditions in urban contexts.

The observed urban–rural differences emphasize that the factors influencing medication adherence are context‐specific. In urban areas, where healthcare services are more accessible, adherence was mainly shaped by wealth and diabetes status, with richer households adhering more and diabetes lowering adherence. In rural areas, adherence was driven by education, wealth, and diabetes, where wealth increased adherence but primary and secondary education paradoxically lowered it, and diabetes again reduced adherence. These findings emphasize the importance of designing public health strategies that respond to the specific needs of each population group. In urban areas, interventions should prioritize simplifying treatment plans and providing integrated support for patients with diabetes and other chronic conditions, alongside strengthening medication literacy through culturally appropriate initiatives. In rural areas, policies must focus on expanding financial support mechanisms and ensuring reliable access to essential medications, with particular attention to economically disadvantaged households and women facing compounded barriers, while also addressing the paradoxical role of education through qualitative, community‐based health literacy programs.

Although this study identified several significant predictors, the Nagelkerke R^2^ values indicate that a considerable proportion of variability in medication adherence remains unexplained. This may be attributed to the use of secondary data, which limited the inclusion of important factors such as patients’ knowledge and awareness of hypertension, medication beliefs, health literacy, physician–patient communication, treatment‐related factors (e.g., side effects and pill burden), and psychosocial influences. These factors are likely to play a crucial role in adherence behavior and should be considered in future research.

## 5. Conclusion

This study offers a detailed evaluation of medication adherence among hypertensive patients in Bangladesh to illuminate the critical impacts of gender, urban–rural divides, socioeconomic status, education, and diabetes on adherence patterns. The findings reveal that urban males demonstrate lower adherence, particularly influenced by wealth and diabetes status. Among females, rural women exhibit reduced adherence associated with educational attainment, while in rural settings overall, wealth emerges as the predominant determinant of adherence behavior. These insights suggest the need for addressing economic disparities in urban areas and treatment simplification for those with comorbidities. In rural regions, expanding financial assistance, educational programs, and ensuring consistent access to medications are essential to support adherence.

## 6. Limitations and Further Scopes

This study has several limitations. First, the use of secondary data from the BDHS restricted the inclusion of potentially important variables such as patients’ knowledge of hypertension, medication beliefs, health literacy, physician–patient communication, and treatment‐related factors. Second, the cross‐sectional design limits causal inference, as it cannot capture changes in adherence behavior over time. Third, medication adherence was based on self‐reported measures, which may be subject to recall and social desirability bias. Excluding cases with missing data may also have introduced selection bias, and the absence of sensitivity analyses limits the ability to assess its impact. Despite these limitations, the study provides nationally representative evidence on factors associated with adherence among hypertensive patients.

To strengthen future research, longitudinal studies should be conducted to assess changes in adherence behavior over time and improve causal inference. Incorporating qualitative methods could offer deeper insights into the sociocultural and behavioral dynamics underlying adherence, particularly among subgroups such as wealthier urban males and educated urban females, where unexpected patterns were observed. Expanding datasets to include variables related to health literacy, care accessibility, and patient–provider interactions would enhance explanatory power. Evaluating the impact of targeted interventions, such as medication literacy programs, simplified treatment protocols, and financial support mechanisms, could inform more effective and equitable public health strategies for managing hypertension in Bangladesh.

## Funding

No funding was received for this manuscript.

## Conflicts of Interest

The authors declare no conflicts of interest.

## Data Availability

The data underlying this study were obtained from the Bangladesh Demographic and Health Survey (BDHS) 2022, conducted under the authority of the National Institute of Population Research and Training (NIPORT), Ministry of Health and Family Welfare, Bangladesh. Access to the BDHS dataset can be requested through the DHS Program website, subject to registration, approval, and compliance with ethical and confidentiality requirements.
